# A Novel Photo-Thermal-Electric Conversion System with an Integrated Support Material

**DOI:** 10.3390/nano13081301

**Published:** 2023-04-07

**Authors:** Peng Kang, Florian Ion Tiberiu Petrescu, Yao Wu, Ying Li, Xin Li, Likui Wang, Gang Shi

**Affiliations:** 1 Key Laboratory of Synthetic and Biotechnology Colloids, Ministry of Education, School of Chemical and Material Engineering, Jiangnan University, Wuxi 214122, China; 2 Department of Mechanisms and Robots Theory, Bucharest Polytechnic University, 060042 Bucharest, Romania

**Keywords:** solar water evaporation, thermoelectricity, integrated device, heat transfer

## Abstract

In conventional photo-thermal-electric conversion systems, the photo-thermal conversion module is coupled to a thermoelectric conversion module. However, the physical contact interface between the modules causes serious energy loss. In order to solve this problem, a novel photo-thermal-electric conversion system with an integrated support material has been developed, with a photo-thermal conversion component at the top, an inside thermoelectric conversion component, and a cooling component at the bottom, surrounded by a water conduction component. The supporting materials of each part are polydimethylsiloxane (PDMS), and there is no apparent physical interface between each part. This integrated support material reduces the heat loss caused by the mechanically coupled interfaces in traditional components. In addition, the confined edge 2D water transport path effectively reduces the heat loss due to water convection. Under 1 sun irradiation, the water evaporation rate and open-circuit voltage of the integrated system reach 2.46 kg m^−2^ h^−1^ and 30 mV, respectively, and are nearly 1.4 times and 5.8 times higher than those of non-integrated systems.

## 1. Introduction

The sun is the Earth’s largest source of light and heat [1], and thermoelectric materials offer the possibility of switching between heat and electricity [2]. A solar thermoelectric generator (STEG) can convert sunlight into heat through a photo-thermal conversion material, resulting in a temperature difference between the two sides of the thermoelectric generator [3]. Finally, based on the Seebeck effect, thermal energy is converted into electrical energy [4]. However, some heat is lost to the environment during the process. Reducing heat loss is the key to improving the STEG energy conversion efficiency [5].

Based on solar water evaporation [6] and solar–thermoelectric conversion, various devices for the co-generation of water and electricity have been developed, thereby increasing energy conversion efficiency [7]. The above devices can be divided into two categories according to their structure [8]. In the first category, a solar water evaporator is positioned on the hot side of the thermoelectric generator, and the thermoelectric generator utilizes the waste heat to generate electricity [9]. Ho et al. reported a 3D organic bucky sponge with broadband light-absorptive, heat-insulative, and shape-conformal abilities for efficient photo-thermal water evaporation [10]. The sponge was then placed on the hot side of the thermoelectric generator to generate electricity by utilizing the waste heat of the sponge in the evaporation process. Simultaneous photo-thermal water evaporation and thermoelectric power generation enhance solar energy utilization efficiency. Lu et al. prepared a layered hydrogel for photo-thermal conversion and water evaporation by impregnating melamine foam into a gel containing porous C/TiC nanohybrids. Due to the efficient light absorption, the photo-thermal conversion, and the heat dissipation by the radiating fin, the synergistic system achieved a high evaporation rate of 0.93 kg m^−1^ h^−1^ and a stable voltage of up to 150 mV under 1 sun irradiation [11]. Cui et al. prepared a novel solar absorber material based on the donor–acceptor type organic small molecule. This material was coated on cellulose filter paper and combined with a commercial thermoelectric generator to form a multifunctional device for simultaneous water evaporation and power generation. Under 1 sun irradiation, the system exhibited stable photo-thermal behavior with an efficiency of 73.98%. Voltages of up to 83 mV, generated by the water flow, can even drive small fans [12]. The second type covered the hot sides of the thermoelectric generator with photo-thermal materials and then placed the water evaporator in direct contact with the cold side of the thermoelectric generator. The waste heat from the cold side of the thermoelectric generator provided the energy for water evaporation [13]. Mu et al. designed a co-generation system based on a thermoelectric generator with a starch–polyacrylamide hydrogel. The hydrogel on the cold side of the thermoelectric generator uses the waste heat for evaporation, which constitutes a multi-stage utilization of solar energy. Moreover, by optimizing the cold-side heat transfer channel, the temperature of the evaporation surface drops below the ambient temperature and can obtain net energy from the environment, further promoting energy utilization [14]. Shi et al. reported a photo-thermal-electric conversion system consisting of an energy storage photo-thermal layer, a thermoelectric generator, and a cooling layer with asymmetric hydrophobicity [15]. The photo-thermal layer is capable of photo-thermal conversion, energy storage, and heat transfer. The system can generate electricity continuously in the dark due to its energy storage capacity. Meanwhile, the cooling layer with asymmetric hydrophobicity has excellent waste energy utilization capability for interfacial water evaporation.

However, in the above work, there is a physical contact interface between the water evaporator and the thermoelectric generator, which can cause heat loss and thus reduce solar utilization efficiency [16]. Therefore, how to reduce the heat loss generated by the physical contact interface needs to be addressed [17]. In this study, an integrated device with photo-thermal and thermoelectric conversions is designed. There is no physical contact interface between the functional units of the device because the support is made from the same material. Finally, this integrated device is applied to water evaporation and power generation.

## 2. Materials and Methods

### 2.1. Materials

Polydimethylsiloxane (PDMS) prepolymer, Zinc oxide (ZnO), Sodium citrate, and Sodium chloride (NaCl) were purchased by Sinopharm Chemical Reagent Co., LTD., Shanghai, China. Boron nitride (BN) was purchased by Innochem Technology Co., LTD., Beijing, China. Multi-layer graphene (G) was supplied by Suzhou Tanfeng Graphene Technology Co., Suzhou, China.

### 2.2. Fabrication of Photo-Thermal Layer

Subsequently, 1.5 g of PDMS prepolymer and 0.15 g of curing agent were mixed and stirred for 15 min. 4.5 g of sodium citrate, a porogenic agent, and a certain amount of graphene (0.02, 0.04, 0.06, 0.08, 0.10 g) were added and stirred for another 15 min. Finally, a portion of the precursor was spread on the bottom of a mold with a diameter of 4 cm and a height of 4 cm (noted as mold-1), and its height was adjusted to 0.5 cm to form a photo-thermal layer (G-PDMS).

### 2.3. Fabrication of Water Transfer Layer

A mixture containing 1.5 g of PDMS prepolymer and 0.15 g of curing agent was stirred for 15 min, then 4.5 g of sodium citrate, a porogenic agent, was added and stirred for 15 min. Then, a hollow cylindrical partition (mold-2) with a diameter of 3.6 cm and height of 4 cm was placed at the center of mold-1, and a portion of the above precursor was filled in the interlayer between mold-2 and mold-1 to form a water transfer layer (PDMS). After curing, mold-2 was removed to create a G-PDMS/PDMS with an internal cavity.

### 2.4. Fabrication of Thermoelectric Layer

Subsequently, 4.5 g NaCl, a certain amount of ZnO, and graphene (the mass fraction of graphene is controlled to be 2.5, 7.5, 10, 15, or 20 wt%), and the content of graphene to be 0.05, 0.10, 0.15, 0.20, or 0.25 g) were added and continually stirred for 15 min. Finally, a portion of the above precursor was filled into the cavity of G-PDMS/PDMS, and its height (with photo-thermal layer) was adjusted to 1.5, 2.0, 2.5, 3.0, or 3.5 cm, thus forming G-PDMS/PDMS/ZnO@G-PDMS with a thermoelectric layer, abbreviated as G/ZnO@G.

### 2.5. Fabrication of Heat Dissipation Layer

A mixture containing 1.5 g of PDMS prepolymer and 0.15 g of curing agent was stirred for 15 min. Next, 4.5 g sodium citrate and a certain amount of boron nitride (0.25, 0.50, 0.75, 1.0, or 1.5 g) were added, and the mixture was continually stirred for 15 min. Then, a part of the above precursor was covered on the surface of G-PDMS/PDMS/ZnO@G-PDMS in mold-1, and the overall internal height was kept consistent with template-1, and the heat dissipation layer (BN-PDMS) was formed after curing. Finally, the samples were soaked in ethanol to remove the porogenic agent to obtain the photo-thermal electrical devices (G-PDMS/PDMS/ZnO@G-PDMS/BN-PDMS, abbreviated as G/ZnO@G/BN)

### 2.6. Water Evaporation and Thermoelectric Performance Tests

An electronic balance was used to measure the change in water mass to measure the evaporation rate. In short, the photo-thermal-electric-water evaporation system was placed above the brine, with the heat dissipation layer in contact with the brine. A xenon lamp was placed at the top of the device to simulate sunlight for the water evaporation experiments, while the ambient temperature and humidity were 23 °C and 17%, respectively. A copper sheet was inserted into the upper and lower poles of the thermoelectric device, and then an electrochemical workstation CHI660 was used to record the open-circuit voltage of the device.

### 2.7. Characterization

Scanning electron microscopy (SEM, S-4800, Hitachi, Tokyo, Japan) was used to observe the surface morphology of samples. X-ray powder diffraction (XRD, Bruker AXS D8, Karlsruhe, Germany) was used to test the crystal patterns of the samples. The voltage and current of the device were measured using the electrochemical workstation (CHI660E). The reflection and spectral absorption of the samples were measured by a UV-Vis NIR spectrophotometer (UV-3600plus, Shimazu Company, Kyoto, Japan). The surface temperature of the samples was measured using an infrared camera (FLIR E6, Portland, OR, USA).

## 3. Results and Discussion

### 3.1. Component Materials and Structure of Photo-Thermal-Electric Conversion Devices

**Figure 1:**
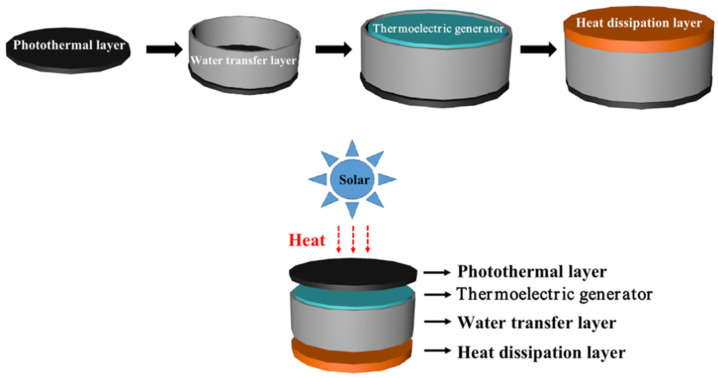
Schematic diagram of the composition of the integrated photo-thermal-electric conversion device.

Figure 1 shows an integrated, flexible device with photo-thermal and thermoelectric conversion, consisting of a photo-thermal conversion layer at the top, a water transfer layer at the periphery, a thermoelectric conversion layer inside, and a thermal dissipation layer at the bottom. All layers are integrated by PDMS as the support material, and the detailed fabrication process has been described in the experimental section. The photo-thermal layer (G-PDMS) comprises a porous support PDMS and a photo-thermal agent graphene (G), converting solar energy into heat and causing water to evaporate while transferring waste heat to the thermoelectric layer below for electricity generation [18]. The water transport layer comprises a porous support PDMS distributed around the device that can transfer water to the photo-thermal layer by capillary force. This confined edge 2D water transport path can effectively reduce heat loss due to water convection [19]. The thermoelectric layer (ZnO@G-PDMS) is composed of non-porous support PDMS, thermoelectric material ZnO, and conductive material graphene (G), which can generate thermoelectric power by the temperature difference between its hot and cold sides. Meanwhile, the non-porous PDMS can avoid contact between the thermoelectric material and the surrounding water to ensure thermoelectric conversion. The thermal dissipation layer (BN-PDMS) is composed of porous support PDMS and thermally conductive material boron nitride (BN), which can effectively reduce the temperature of the cold side of the thermoelectric layer [20]. This integrated device can effectively minimize heat loss by the mechanically coupled interfaces in the traditional non-integrated device to improve solar energy utilization efficiency.

**Figure 2:**
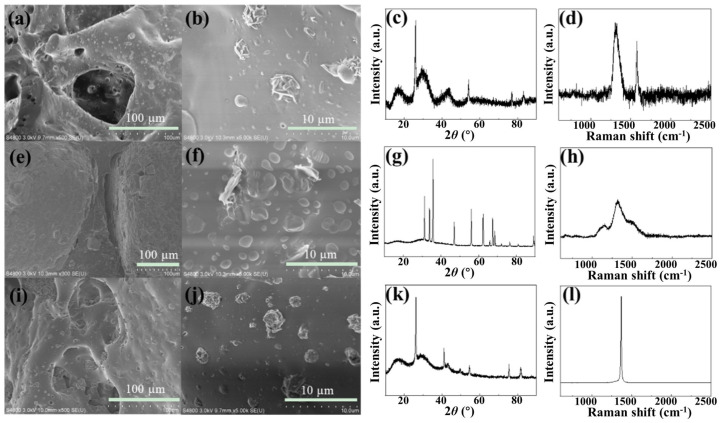
SEM images of different parts of the device. (**a**,**b**) Photo−thermal layer G−PDMS; (**e**,**f**) Thermoelectric layer ZnO@G−PDMS; (**i**,**j**) Dissipative layer BN−PDMS. XRD patterns: (**c**) G−PDMS; (**g**) ZnO@G−PDMS; (**k**) BN-PDMS; Raman patterns: (**d**) G−PDMS; (**h**) ZnO@G PDMS; (**l**) BN−PDMS.

Figure 2a,b show the SEM images of the photo-thermal layer (G-PDMS) at different resolutions and demonstrate that the pore size of PDMS with a 3D mesh structure is on the micron scale. The graphene nanosheets were embedded into PDMS to increase its surface roughness. The successful mixing of graphene and PDMS was demonstrated by XRD [21] and Raman [22] characterization of G-PDMS (Figure 2c,d). Figure 2e,f show that the graphene nanosheets and ZnO nanoparticles are embedded into the thermoelectric layer (ZnO@G-PDMS) and PDMS, and this part was dense and non-porous. XRD [23] and Raman [24] characterization of ZnO@G-PDMS (Figure 2g,h) indicates that graphene and ZnO were successfully mixed into the PDMS support. Figure 2j shows the SEM images of the thermal dissipation layer (BN-PDMS) and the pore size of PDMS with a 3D mesh structure at the micron scale. XRD [25] and Raman [26] characterization of BN-PDMS (Figure 2k,l) proved the presence of boron nitride.

### 3.2. Optimizing the Performance of Photo-Thermal-Electrical Devices

**Figure 3:**
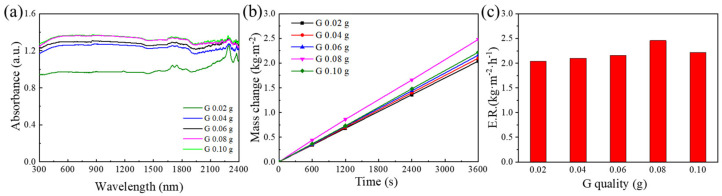
(**a**) UV−vis NIR absorption spectra; (**b**) Evaporation mass variation; (**c**) Evaporation rates of photo−thermal layers with different graphene contents.

To investigate the effect of photo-thermal material content on the water evaporation rate of photo-thermal-electric devices, the performance of the devices with different amounts of G added to their photo-thermal layers was compared. Figure 3a shows the UV-vis NIR absorption spectra of the photo-thermal layers containing different G additions. The light absorption capacity gradually increased with the addition of G. Light absorption is more efficiently converted into heat for evaporation, thereby increasing the evaporation rate. When the addition of G was 0.08 g, the light absorption capacity reached its maximum. The continuous increase in the absorber concentration resulted in almost no enhancement in solar absorption but an aggregation of G, leading to some blocking of pores. This prevented heat transfer to the water and increased heat loss. Therefore, the evaporation rate increased and then decreased with adding G, as shown in Figure 3b,c. The maximum value for device evaporation of 2.46 kg m^−2^ h^−1^ was obtained when 0.08 g of G was added.

**Figure 4:**
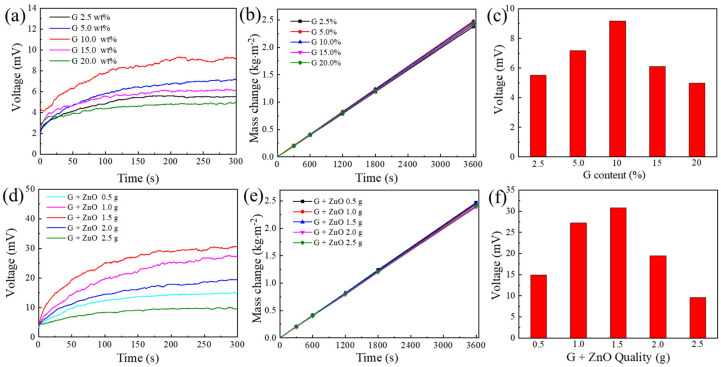
(**a**) Open−circuit voltage; (**b**) Evaporation mass change; (**c**) Maximum open−circuit voltage of the device for changing the proportion of graphene in the thermoelectric layers. (**d**) Open−circuit voltage; (**e**) Evaporation mass change; (**f**) Maximum open−circuit voltage of the device for changing the total amount of graphene and ZnO in the thermoelectric layers.

Figure 4a–c highlight the effect of the ratio of G and ZnO in the thermoelectric layer on the thermoelectricity and water evaporation of the device for a certain total amount of G and ZnO. As the proportion of G increased, the open-circuit voltage of the device increased and then decreased. Since ZnO is a broadband semiconductor material with poor electrical conductivity and high resistance [27], adding G with excellent conductivity should improve the thermoelectric properties of the device [28]. However, when the proportion of G is too high, the number of carriers generated decreases due to insufficient ZnO, and thus the thermoelectric performance of the device decreases. In addition, the proportion of G in the thermoelectric layer had almost no effect on the evaporation rate of the device. Therefore, the optimal value of the percentage of G is 10 wt%. Figure 4d–f show the effect of changing the total amount of G and ZnO on the thermoelectric and evaporation performance of the device for a G percentage of 10 wt%. The device performed best when the total amount of G and ZnO was 1.5 g.

**Figure 5:**
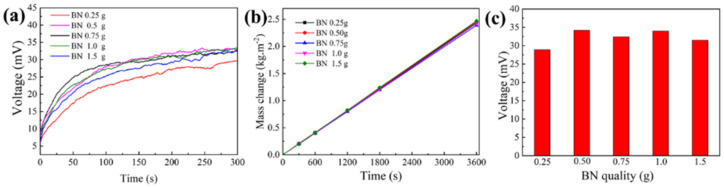
(**a**) Open circuit voltage; (**b**) Evaporation mass variation; (**c**) Maximum open−circuit voltage for different BN amounts in the cooling layer of the thermoelectric component.

Figure 5 highlights the effect of different BN additions in the cooling layer on the device’s thermoelectric and water evaporation performance. As shown in Figure 5a,c, the open-circuit voltage gradually increased and stabilized with increasing BN addition. The open-circuit voltage reached its maximum when the BN addition was 0.5 g. The high thermal conductivity of the dissipation layer removed the most heat from the cold end of the thermoelectric layer because the BN content enhanced the dissipation layer’s thermal conductivity. The increase in temperature difference between the hot and cold ends of the thermoelectric layer enhanced the thermoelectric performance of the device. The open-circuit voltage of the device did not increase further when the BN addition exceeded 0.5 g due to the thermal conductivity of the dissipation layer reaching its limit. Figure 5b illustrates that the addition of BN had a negligible effect on the evaporation rate of the device. Therefore, the optimal amount of BN in the cooling layer was 0.5 g.

### 3.3. Mechanism Analysis

**Figure 6:**
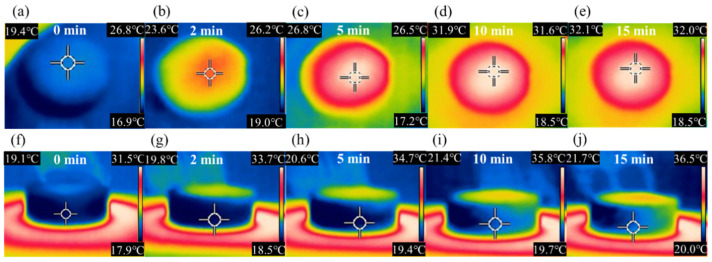
Infrared images of (**a**–**e**) the top of the device and (**f**–**j**) the cold end of the thermoelectric layer of the device at different illumination times.

Figure 6 shows the infrared images of the surface and the cold side of G/ZnO@G/BN analyzed under 1 sun irradiation. Figure 6a–e show that the surface temperature of G/ZnO@G/BN gradually increased with the illumination time. The initial surface temperature was 19.4 °C and was then stabilized at ~32 °C after 10 min of illumination. Figure 6f–j show the infrared images of the cold side of G/ZnO@G/BN, where the initial temperature was 19.1 °C. The temperature at the cold side then reached stability (21.4 °C) after 10 min. At this point, the device was in heat transfer equilibrium. The integrated design of the device and the heat sink layer doped with highly thermally conductive materials enabled rapid heat transfer from the cold end of the thermoelectric layer to the water below. The temperature difference between the hot and cold ends of the thermoelectric layer increased, and the thermoelectric power generation efficiency improved.

**Figure 7:**
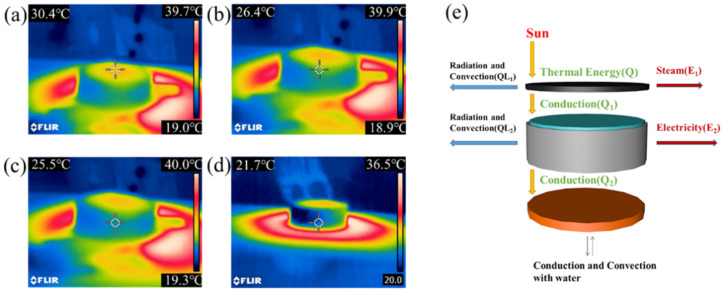
(**a**–**d**) Top-down infrared thermal images of the thermoelectric device; (**e**) Heat transfer mechanism of the thermoelectric device.

Figure 7a–d show the working temperature at different locations of the device G/ZnO@G/BN recorded by the infrared camera. The surface temperature of the photo-thermal layer, the maximum temperature of the thermoelectric layer, the intermediate temperature of the thermoelectric layer, and the minimum temperature of the thermoelectric layer were 30.4 °C, 26.4 °C, 25.5 °C, and 21.7 °C, respectively, with a decreasing temperature trend. Figure 7e analyzes the mechanism of heat transfer for the overall device. Assuming that the energy of incident light is E, the heat reaching the photo-thermal layer is *Q*. Part of *Q* is transferred to the air and dissipated through thermal radiation and thermal convection (*QL*_1_), part of Q is used for water evaporation (*E*_1_), and part of *Q* is transferred to the thermoelectric layer through heat conduction (*Q*_1_). Then, part of *Q*_1_ is transferred to the cooling layer by heat conduction (*Q*_2_), part of *Q*_1_ is dissipated by heat radiation and heat convection (*QL*_2_) to the air, and part of *Q*_1_ is used to generate electricity (*E*_2_). After *Q*_2_ is transferred to the cooling layer, it is dissipated through heat exchange with water.

The calculation of solar energy utilization efficiency *Φ*_1_ is shown in the Formulas (1)–(4).

(1) $$\Phi_{1}=(E_{1}+E_{2})⁄E$$

(2) $$E_{1}=MH$$

(3) $$E_{2}=VIt$$

(4) E=Pt

*E* is the total energy from sunlight to the device, and *P* refers to the solar input power. When the power density of 1 sun is 1 kW m^−2^, and the device’s radius is 2 cm, calculated *P* is 1.26 W. *E*_1_ is the energy for water evaporation, *M* is the mass of evaporated water, and *H* is the enthalpy of evaporation. According to the literature, the mass of lost water is 0.257 g when evaporating for 300 s, and *H* is 1255 J g^−1^ [29]. *E*_2_ is the electrical energy generated by the thermoelectric layer, *V* refers to the open-circuit voltage of the whole system, and *I* refers to the short-circuit current of the whole system. As shown in Figure 9a,b, the open-circuit voltage and short-circuit current were 30 mV and 1.1 μA, respectively. Working time *t* was 300 s, and the calculated solar energy utilization efficiency was 86%.

### 3.4. Performance Comparison of Photo-Thermal-Electric Devices

**Figure 8:**
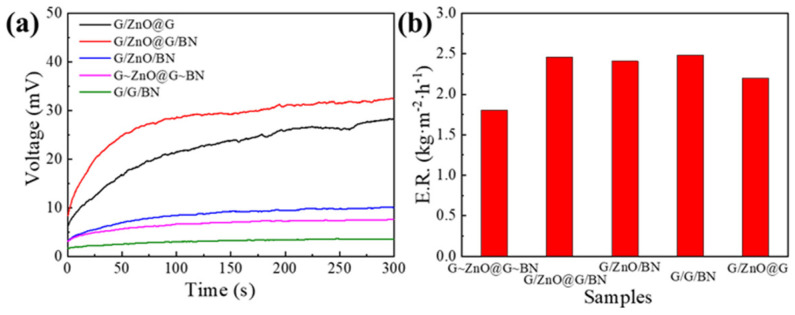
(**a**) Open circuit voltage and (**b**) SVG rate for different devices.

As shown in Figure 8, ZnO and G in the thermoelectric layer, BN in the thermal dissipation layer, and integrated design are crucial to the overall system’s photo-thermal-electrical conversion performance. Firstly, to verify the contribution of ZnO in the thermoelectric layer to the system, the device G/G/BN was fabricated without ZnO and then compared to G/ZnO@G/BN with ZnO for thermoelectricity and water evaporation. The results show that the open-circuit voltage of G/ZnO@G/BN was much higher than that of G/G/BN. The reason is that ZnO, as a thermoelectric material, can convert the temperature difference between the hot and the cold sides of the device into electric energy, and therefore the open-circuit voltage of G/G/BN without ZnO is almost 0 V. Next, to verify the necessity of G doping in the thermoelectric layer on the device performance, G/ZnO/BN was fabricated without G doping, and its performance was compared to that of G/ZnO@G/BN with G doping. The results show that the open-circuit voltage of the G-doped G/ZnO@G/BN was much higher than that of the non-G-doped G/ZnO/BN. The adequate addition of G to the thermoelectric layer can significantly improve its conductivity and thus increase its open-circuit voltage. Then, the contribution of BN in the thermal dissipation layer to the device performance was also verified. The performance of G/ZnO@G/BN with BN was compared to that of G/ZnO@G without BN. The results show that the open-circuit voltage of G/ZnO@G without BN is lower than that of G/ZnO@G/BN. This is because the BN in the thermal dissipation layer is a thermally conductive material, which can accelerate the heat transfer from the cold side and increase the temperature difference between the hot and cold sides of the device. Finally, to verify the advantage of integrated design, a component-based device was fabricated, named G~ZnO@G~BN. In short, the photo-thermal, thermoelectric, water transport, and thermal dissipation layers were fabricated separately in the same size mold and then stacked together. Comparing the performance of the component-based device G~ZnO@G~BN to that of the integrated device G/ZnO@G/BN, G~ZnO@G~BN showed lower thermoelectric performance than that of G/ZnO@G/BN. The mechanical coupling of G~ZnO@G~BN causes discontinuous contact interfaces and heat loss in the transfer process. In addition, the water evaporation performance of each comparison sample was characterized. As shown in Figure 8b, the water evaporation performance of the G/ZnO@G without the thermal dissipation layer and the component-based G~ZnO@G~BN was lower compared to the other devices. The hydrophilic area of G/ZnO@G without a thermal dissipation layer is smaller than that of G~ZnO@G~BN, resulting in a slow and insufficient water transfer to the photo-thermal layer and a lower evaporation rate. In the component-based device G~ZnO@G~BN, non-uniform water transport caused by the non-uniform contact at the interface was observed. The serious water supply shortage leads to a low evaporation rate.

### 3.5. Cyclic Stability

**Figure 9:**
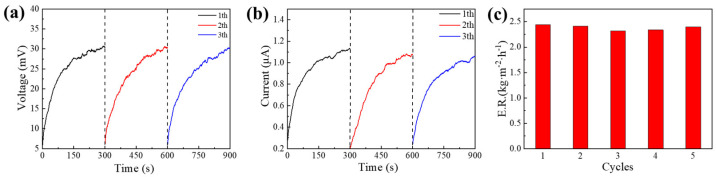
Cycling stability of the device G/ZnO@G/BN. (**a**) Open−circuit voltage; (**b**) Short−circuit current; (**c**) evaporation rates.

Cyclic stability is an important indicator for evaluating photo-thermal-electric conversion systems. Three cycling tests of open-circuit voltage and short-circuit current were performed, as shown in Figure 9a,b. Each cyclic test was conducted under 1 sun irradiation for 300 s. The open-circuit voltage and short-circuit current were almost constant during three testing cycles, indicating that the device had a stable performance. The corresponding open-circuit voltage and short-circuit current were 30 mV and 1.1 µA, respectively. In addition, the interfacial evaporation capability of the device was tested for five cycles, and each cyclic test was conducted under 1 sun irradiation intensity for 2 h, as shown in Figure 9c. The results show that the device exhibits excellent evaporative stability.

## 4. Conclusions

The flexible photo-thermal-electric conversion device was integrated with the photo-thermal conversion layer, the water transfer layer, the thermoelectric conversion layer, and the thermal dissipation layer through the support PDMS. The content of graphene in the photo-thermal layer, the content and ratio of graphene and ZnO in the thermoelectric layer, and the content of BN in the thermal dissipation layer were adjusted, respectively. The optimal device achieves a water evaporation rate of 2.46 kg m^−2^ h^−1^ and an open-circuit voltage of 30 mV under 1 sun irradiation, with a solar energy utilization efficiency of 86%. This integrated design solves the energy loss caused by the mechanical coupling of the traditional photo-thermal and thermoelectric modules.

## Data Availability

Not applicable.
